# The impact of the mRNA COVID-19 vaccine on the Th-like cytokine profile in individuals with no history of COVID-19: insights into autoimmunity targeting heat shock proteins

**DOI:** 10.3389/fimmu.2025.1549739

**Published:** 2025-03-14

**Authors:** Stefan Tukaj, Magdalena Sitna, Krzysztof Sitko

**Affiliations:** Department of Molecular Biology, Faculty of Biology, University of Gdańsk, Gdańsk, Poland

**Keywords:** cytokines, T helper cells, COVID-19, heat shock proteins, SARS-CoV-2, vaccine

## Abstract

Although some reports suggest that COVID-19 vaccination may exacerbate existing autoimmune diseases or trigger new-onset cases, a definitive causal relationship between the vaccines and these conditions has not been established. Several potential mechanisms have been proposed to explain this association, including: (i) molecular mimicry, which refers to a structural similarity between SARS-CoV-2 and human antigens; (ii) bystander activation, involving both B and T lymphocytes; and (iii) the effects of adjuvants. In this study, we investigated whether two doses of the mRNA COVID-19 vaccine influenced blood cytokine levels associated with major T helper cell populations, which are known to play a significant role in autoimmunity and revisited the role of the humoral autoimmune response directed against heat shock proteins (Hsps) in individuals with no history of COVID-19. While no significant differences were found in the levels of IFN-γ, IL-6, IL-22, IL-4, IL-8, IL-10, and IL-17A, between vaccinated and unvaccinated people, several positive correlations were observed between serum cytokine levels and circulating autoantibodies directed against self-Hsps exclusively in vaccinated individuals. These findings suggest that the mRNA COVID-19 vaccine does not impact cytokines involved in the pathogenesis of autoimmune diseases. Further research is required to evaluate the safety of COVID-19 vaccination in patients with autoimmune conditions, particularly those in whom anti-Hsps autoantibodies are suspected to contribute to disease development.

## Introduction

The COVID-19 pandemic, caused by the severe acute respiratory syndrome coronavirus 2 (SARS-CoV-2), emerged in Wuhan, China, in late 2019 and posed significant challenges to healthcare systems worldwide. The clinical manifestations of SARS-CoV-2 infection varied widely, ranging from asymptomatic cases to severe respiratory failure and high mortality rates, particularly among elderly individuals with pre-existing conditions. Beyond respiratory complications, COVID-19 was associated with several extrapulmonary issues, including cardiovascular and cerebrovascular diseases (1–3). A key focus of COVID-19 research has been understanding the factors driving the variability in disease severity and immune responses among individuals. Considerable efforts have been dedicated to exploring the cellular mechanisms underpinning SARS-CoV-2-induced immune responses, with the goal of identifying novel biomarkers, prognostic tools, and therapeutic targets. Early findings highlighted the critical role of cytokines in the progression of COVID-19. Analyzing qualitative, quantitative, and temporal differences in cytokine expression has become vital to combating the disease. Cytokines have emerged as central players in the pathogenesis of COVID-19, acting as prognostic indicators of the disease severity and outcomes. SARS-CoV-2-induced cytokine expression has been shown to significantly disrupt immune regulation, triggering autoinflammation, organ failure, and even death. Critically ill COVID-19 patients frequently exhibit a cytokine storm, characterized by elevated levels of cytokines such as IL-6, IL-8, IL-12, TNFα, IL-17, MCP-1, IP-10, and IL-10. This surge in cytokine levels contributes to immune imbalance, heightened inflammatory responses, infiltration of neutrophils and macrophages, and subsequent lung damage (4–6). Autoimmune and inflammatory conditions have been broadly associated with numerous infectious diseases, including COVID-19 (7, 8). Several hypotheses have been put forward to elucidate the molecular mechanisms underlying immune dysregulation in COVID-19. These include molecular mimicry by viral proteins, the systemic nature and multiorgan impact of COVID-19 linked to the widespread expression of the ACE2 receptor for SARS-CoV-2, bystander activation of immune cells, the release of autoantigens from virus-damaged tissues, superantigen-driven lymphocyte activation, and epitope spreading (8). Studies comparing the immune responses in COVID-19 and autoimmune disorders have concluded that immune-mediated mechanisms are largely responsible for organ damage in COVID-19, paralleling autoimmune disease processes. Furthermore, various autoantibodies commonly observed in autoimmune diseases such as antinuclear antibodies, lupus anticoagulant, cold agglutinins, and anti-Ro/SSA antibodies, have been detected in COVID-19 patients (9). It is hypothesized that both viral and bacterial receptors work together to drive the increased inflammation required for the development of autoimmune diseases. SARS-CoV-2 primarily activates innate receptors associated with viruses, including TLR3, TLR7, TLR8, NLRP3, RIG-1, and MDA-5. However, severe COVID-19 is marked by the additional activation of receptors such as TLR1, TLR2, TLR4, TLR5, TLR6, NOD1, and NOD2, which are mainly responsive to bacterial antigens. The activation patterns of the innate immune system seen in autoimmune coagulopathies, myocarditis, Kawasaki disease, and multisystem inflammatory syndrome in children are like those observed in severe COVID-19 rather than in SARS-CoV-2 infection alone, suggesting that autoimmunity may follow a combined viral and bacterial infection (10). Furthermore, the microbiome appears to influence the progression of long COVID-associated autoimmunity, including the severity of illness, recovery rate, and the onset of autoimmune reactions. While the exact role of the microbiome in long COVID autoimmunity remains under study, recent research suggests that interventions targeting the microbiome, such as probiotics, prebiotics, and dietary modifications, could potentially reduce autoimmune responses and improve long-term outcomes for COVID-19 survivors (11).

One of the most significant advancements in combating COVID-19 has been the development of vaccines that elicit a specific immune response against SARS-CoV-2. Recent breakthroughs in mRNA-based technologies have facilitated the rapid development and deployment of new therapeutics, marking a new era in medicine. These mRNA-based vaccines enable robust and transient protein expression without entering the nucleus or posing a risk of genomic integration, making them versatile tools for treating or preventing a wide range of conditions, including infectious diseases, cancer, and genetic disorders (12). Shortly after the pandemic’s onset, COVID-19 vaccines were introduced as essential tools for controlling the spread of the virus and mitigating its impact. Clinical studies and systematic reviews have largely supported the favorable safety profiles of mRNA vaccines. However, concerns have emerged regarding potential adverse events and the long-term safety of these vaccines. Evidence suggests that COVID-19 vaccination may affect immune regulation and, in rare cases, lead to autoimmune disorders, such as autoimmune glomerulonephritis, autoimmune rheumatic diseases, and autoimmune hepatitis. Severe complications reported included: thrombotic thrombocytopenia, vaccine-induced immune thrombotic thrombocytopenia, myocarditis, pericarditis, Guillain-Barré syndrome, Bell’s palsy, neuromyelitis optica spectrum disorder, multiple sclerosis, and vitiligo (13–16). Currently, there is insufficient evidence to determine whether the rare occurrence or exacerbation of autoimmune bullous diseases (AIBDs) following infection with SARS-CoV-2 or vaccination against it represents a specific immunopathology driven by this virus, or merely a nonspecific immune dysregulation like that seen with other infectious diseases. Vaccination against COVID-19 in AIBD patients has been linked to a low incidence of minor disease flares while providing protection against severe COVID-19 (17, 18). Additional research is needed to clarify the potential causal link between COVID-19 vaccines and autoimmune diseases.

Previous research, including our own, has highlighted a potential role for autoantibodies targeting self-heat shock proteins (Hsps) in the development of autoimmunity (19) with prior hypothesis suggesting that circulating anti-SARS-CoV-2 IgG antibodies generated during vaccination or infection might cross-react with human Hsps due to molecular mimicry, potentially triggering autoimmune responses (20). However, our earlier findings challenged this assumption, demonstrating that anti-Hsp60/70/90 autoantibody levels remain unchanged in individuals with anti-SARS-CoV-2 IgG antibodies induced by vaccination or infection (21). Nevertheless, SARS-CoV-2 relies on host molecular chaperones, particularly Hsp90 and Hsp70, to facilitate the processes of entry and replication. Research has shown that Hsp90 is overexpressed in the damaged lungs of COVID-19 patients, and Hsp90 inhibitors have been found to prevent and repair pulmonary microvascular damage caused by the SARS-CoV-2 spike protein. Additionally, inhibition of Hsp90 activity has been shown to reduce both SARS-CoV-2 replication and the expression of pro-inflammatory cytokines in primary human airway epithelial cells (22). Given the information above, it is crucial to further investigate the role of immunogenic heat shock proteins (Hsps), whose involvement in the development of autoimmune diseases has been well-established (19, 23), in the activation of the post-vaccination immune response.

In this study, we investigated whether two doses of the mRNA COVID-19 vaccine influenced blood cytokine levels associated with major T helper cell populations, which are known to play a significant role in autoimmunity. We also revisited the role of the humoral autoimmune response directed against heat shock proteins (Hsps) in the vaccinated individuals with no history of COVID-19, as these molecules are suspected to play a significant role in autoimmune diseases. Our aim was to investigate whether association patterns between Th-like cytokine levels and anti-HSP autoantibodies differ between unvaccinated and vaccinated individuals, and whether the latter group exhibits any similarities to patients suffering from autoimmune diseases.

## Results

### The mRNA COVID-19 vaccine does not influence the Th-like cytokine profile in individuals with no history of COVID-19

**Figure 1:**
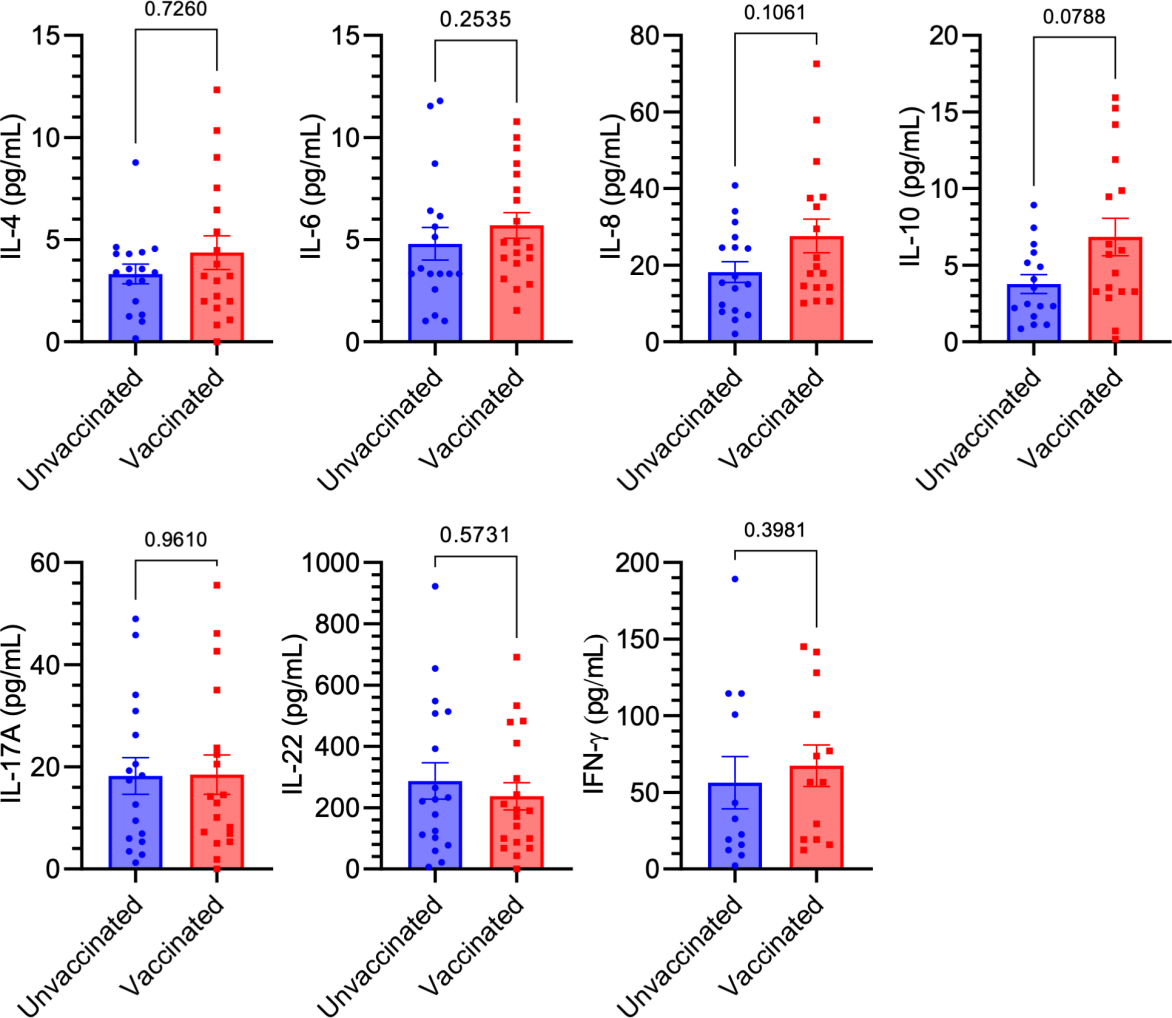
Comparison of Th-like cytokine levels between vaccinated and unvaccinated individuals. No significant differences were observed in the serum levels of IFN-γ, IL-6, IL-22, IL-4, IL-8, IL-10, and IL-17A between anti-SARS-CoV-2 IgG-positive (Vaccinated, n = 20) and anti-SARS-CoV-2 IgG-negative (Unvaccinated, n = 20) individuals, as measured by ELISA. Squares/dots represent individual values, while bars indicate the mean (± SEM) for each group.

In this study, we examined whether two doses of the mRNA COVID-19 vaccine affected blood cytokine levels associated with major T helper cell populations, which are known to play a critical role in autoimmunity (24), in individuals with no history of COVID-19. Serum samples were collected from blood donors between December 2020 and February 2021, comprising either unvaccinated (n = 20) or vaccinated (n = 20) volunteers. All participants were screened for the presence of anti-SARS-CoV-2 IgG targeting the S1 domain of the viral spike protein using a semiquantitative ELISA test. Vaccinated participants were additionally assessed for circulating anti-SARS-CoV-2 IgG S1 levels using a quantitative ELISA assay within 3–5 weeks of their final vaccine dose. All vaccinated individuals tested positive for anti-SARS-CoV-2 IgG against the S1 domain, whereas all unvaccinated participants were negative for this marker. Using a panel of ELISA tests, we observed no significant differences in the levels of IFN-γ, IL-6, IL-22, IL-4, IL-8, IL-10, and IL-17A between vaccinated and unvaccinated people. However, a trend (p = 0.0788) toward increased levels of the anti-inflammatory cytokine IL-10 was observed in vaccinated participants compared to their unvaccinated counterparts (**Figure 1**).

### Th-like cytokine profile correlates with circulating autoantibodies directed to Hsps exclusively in mRNA COVID-19 vaccinated people with no history of COVID-19

**Figure 2:**
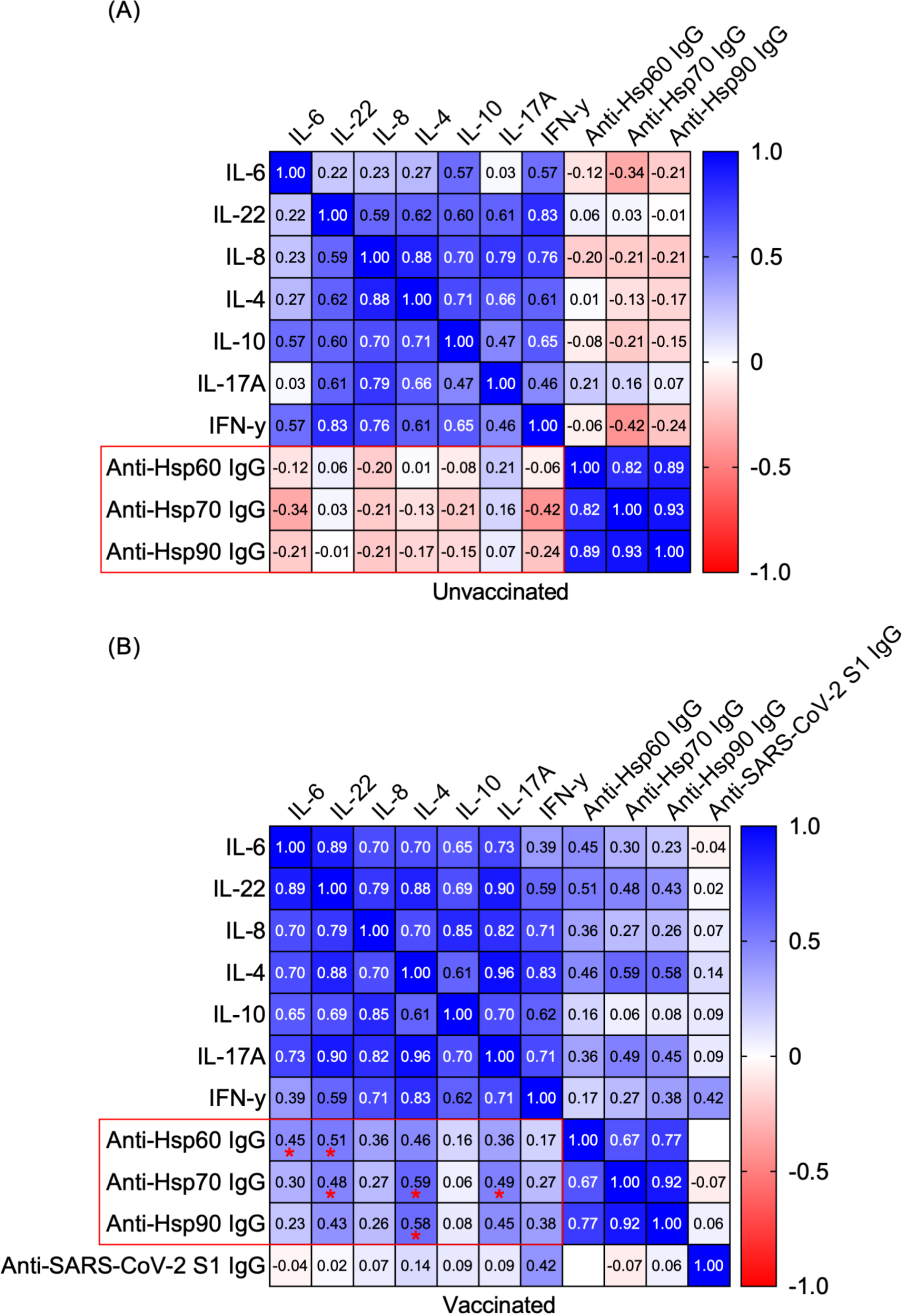
Positive correlations between serum cytokines and circulating autoantibodies against self-Hsps were observed exclusively in vaccinated individuals. Heatmap matrix displaying Spearman’s rank correlation coefficients for the relationships between serum levels of IL-6, IL-22, IL-4, IL-8, IL-10, IL-17A, IFN-γ and circulating anti-Hsp IgGs or anti-SARS-CoV-2 IgG S1. Comparisons were conducted within the groups: **(A)** anti-SARS-CoV-2 IgG-negative (Unvaccinated) and **(B)** anti-SARS-CoV-2 IgG-positive (Vaccinated). Correlation coefficients (r values) are shown within the heatmap boxes, with statistically significant correlations highlighted in red frames and marked with asterisks (*P < 0.05).

In this study, we revisited the role of the humoral autoimmune response targeting autologous Hsps in mRNA COVID-19-vaccinated individuals with no history of COVID-19. Specifically, we investigated its associations with various cytokines (i.e., IFN-γ, IL-6, IL-22, IL-4, IL-8, IL-10, and IL-17A) in both vaccinated and unvaccinated people. A multivariable non-parametric Spearman rank correlation analysis identified several positive associations between serum cytokine levels (i.e., IL-6, IL-22, IL-4, and IL-17A) and circulating autoantibodies against self-Hsp60/70/90 IgG, exclusively in vaccinated individuals (**Figure 2**). While a set of positive mutual correlations among the analyzed cytokines and anti-Hsps autoantibodies was observed regardless of anti-SARS-CoV-2 IgG antibody positivity (**Figure 2**), no significant correlations were identified between anti-Hsp60/70/90 IgG autoantibody or cytokine levels and circulating anti-SARS-CoV-2 IgG antibody levels in vaccinated individuals (**Figure 2**). All scatter plots and correlation analyses are provided in **Supplementary Data 1**.

## Discussion

The COVID-19 pandemic has posed a significant global challenge in recent years, marked by high rates of illness and death. While vaccination has played a crucial role in controlling the spread of the virus and mitigating the impact of the disease, concerns about its safety and potential side effects have emerged. Among the most widely administered vaccines were Pfizer-BioNTech, followed by AstraZeneca and Sinovac. Over three-quarters of participants reported experiencing side effects after the first dose, with the majority being mild and localized, such as pain and redness at the injection site (25). In this study, we investigated whether two doses of the mRNA COVID-19 vaccine influenced blood cytokine levels associated with major T helper cell populations. These cells are a heterogeneous group of immune cells that play central roles in nearly all aspects of immune responses. T helper cells are activated through peptide-MHC class II complexes on antigen-presenting cells (APCs), costimulatory signals, and cytokine signaling, differentiating into various subsets characterized by distinct surface molecules, cytokines, and key CD4+ markers. These subsets include Th1, Th2, Treg, follicular helper T cells, Th17, Th9, Th22, and CD4+ cytotoxic T lymphocytes (17). Specifically, we compared the levels of IFN-γ, IL-6, IL-22, IL-4, IL-8, IL-10, and IL-17A between anti-SARS-CoV-2 IgG-positive and seronegative individuals, all without a history of COVID-19. We found no significant differences in the analyzed cytokine levels between vaccinated and unvaccinated individuals. However, a trend toward increased levels of the anti-inflammatory cytokine IL-10 was observed in vaccinated participants compared to unvaccinated ones. This trend is particularly beneficial, as recent studies have linked IL-10 to the severity and mortality of patients with acute or post-acute SARS-CoV-2 infection. IL-10 acts as an endogenous “danger signal,” released in response to the peak of circulating pro-inflammatory cytokines, helping to protect the body from damage caused by a harmful hyperinflammatory state (26). Furthermore, numerous studies in autoimmune diseases have shown altered IL-10 serum levels, suggesting a potential connection between IL-10 and disease progression. Research in mice further indicates that IL-10 production may play a protective role in organ-specific autoimmune diseases by regulating the balance between pathogenic Th1 cells and protective, anti-inflammatory Th2 cells (26). The findings of this study challenge the initial hypothesis concerning the impact of mRNA COVID-19 vaccination on the development of autoimmune diseases via mechanisms involving pro-inflammatory cytokines, which are characteristic of T helper cells pivotal to the progression of pathological conditions. On the other hand, our study shows partial alignment with prior research, which demonstrated that, both initially and during recovery from symptomatic COVID-19, fully vaccinated individuals had lower levels of inflammatory markers i.e., IL-2RA, IL-7, IL-8, IL-15, IL-29, IP-10, monocyte chemoattractant protein-1, and TNF-α compared to unvaccinated participants. This suggests that vaccination may be linked to both short-term and long-term reductions in inflammation, which could help explain the decreased severity of the disease and lower mortality rates in vaccinated individuals (27). Another study explored the cytokine and chemokine responses to the first and second doses of the BNT162b2 mRNA vaccine (Pfizer/BioNTech) both in antigen-naive individual and those previously infected with SARS-CoV-2. The researchers found increased levels of IL-15 and IFN-γ shortly after the booster dose, which were correlated with Spike antibody concentrations. A distinct systemic pattern emerged, showing rises in IL-15, IFN-γ, and IP-10/CXCL10 following the first dose, which were further enhanced by TNF-α and IL-6 after the second dose. In individuals with prior COVID-19, a single dose of the vaccine triggered cytokine levels and antibody responses similar to those observed after the booster dose in individuals without previous antigen exposure (28). Another study highlights the coordinated immune responses to the BNT162b2 mRNA vaccine and underscores the critical role of a network of innate immune responses, particularly IL-15, in shaping the adaptive immune response following vaccination (29). While the authors also observed increased levels of cytokines such as TNF-α, IFN-γ, IL-6, and IL-10 in response to the mRNA vaccine for anti-COVID-19 (29), in our study, the levels of these cytokines (except for IL-10) did not change significantly. This discrepancy may largely be attributed to the fact that, unlike in our analysis, the cytokine analysis in the aforementioned study was conducted 24 hours after the second vaccine dose.

Based on these observations, it can be concluded that a key factor in cytokine induction in individuals receiving the COVID-19 vaccine is the time interval between the vaccine dose and serological analysis, the number of doses administered, and whether the analysis was performed in individuals with a history of COVID-19. It is worth to note, that the cytokine signature associated with an effective anti-COVID-19 response may differ in immunocompromised individuals. For example, in patients with hematological malignancies who failed to produce anti-SARS-CoV antibodies, the innate systemic response was primarily characterized by IL-8 and MIP-1α, with a significant reduction in the IFN-γ, IL-15, and IP-10/CXCL10 response (30).

According to our analyses, we can infer that the administration of two doses of the mRNA COVID-19 vaccine does not have a lasting impact on the production of pro-inflammatory cytokines typically associated with T helper cell populations. However, additional follow-up studies at various time intervals should be conducted using a larger cohort of participants.

Several studies have suggested that molecular mimicry between immunogenic proteins of SARS-CoV-2 and human molecules may contribute to the development of autoimmunity (31, 32). Marino Gammazza et al. (2020) proposed that SARS-CoV-2 could potentially trigger an autoimmune response due to molecular mimicry between human heat shock proteins (Hsps) and immunogenic viral proteins (20). Both bacterial and autologous extracellular Hsps are known to interact with innate and adaptive immune cells, potentially initiating a humoral (auto)immune response and the production of anti-Hsp (auto)antibodies. Based on the assumption that circulating anti-SARS-CoV-2 IgG, generated during vaccination or infection, might cross-react with human Hsps, we hypothesized that volunteers who received the COVID-19 vaccine or had recovered from COVID-19 would show higher titers of anti-Hsp antibodies in their serum. However, our previous research contradicted this hypothesis, demonstrating that levels of anti-Hsp90/70/60 autoantibodies did not change in individuals with anti-SARS-CoV-2 IgG antibodies induced by either vaccination or infection (21). Nevertheless, the potential role of circulating anti-Hsp autoantibodies in modulating cytokine profiles in vaccinated individuals with autoimmune conditions warrants further investigation, given the crucial role of proinflammatory cytokines in these diseases. In addition, higher levels of anti-Hsp autoantibodies are found in patients suffering from numerous inflammatory and autoimmune diseases, including rheumatoid arthritis, juvenile idiopathic arthritis, autoimmune myasthenia gravis, dermatitis herpetiformis, psoriasis, systemic lupus erythematosus, epidermolysis bullosa acquisita, celiac disease, atopic dermatitis and other (auto)inflammatory diseases (19). In this study, we revisited the role of the humoral autoimmune response targeting autologous Hsps in individuals vaccinated with mRNA COVID-19 vaccines who had no prior history of the disease. We identified several positive correlations between serum cytokine levels-such as IL-6, IL-22, IL-4, and IL-17A (but not IL-8, IL-10 or IFN-γ)-and circulating autoantibodies against self-Hsp60/70/90 IgG, observed exclusively in vaccinated individuals.

Notably, we observed a positive correlation (or a strong tendency) between anti-Hsp60/70/90 IgG autoantibodies and IL-4, a key Th2 cytokine involved in initiating and expanding humoral immunity (33). The antagonistic effect of IL-4 on Th1 polarization (a population characterized by IFN-γ production) suggests its potential as a therapeutic agent for autoimmune diseases (33). In contrast, the positive correlations between anti-Hsp60 IgG and IL-6 or IL-22, as well as between anti-Hsp70 IgG and IL-22 or IL-17A, may indicate a proinflammatory activity. The discrepancies in intraindividual correlations between anti-Hsps and cytokines in vaccinated individuals could be attributed to the distinct immune roles of extracellular Hsps. While both intra- and extracellular Hsp90 are typically regarded as initiators of proinflammatory responses, the extracellular forms of Hsp60 or Hsp70 can exhibit either pro- or anti-inflammatory effects. These effects depend on factors such as the source (bacterial or self-derived Hsps), the mode of Hsp secretion (whether via necrosis or active liberation), and the specific immune mechanisms involved in different autoimmune diseases (23, 34). These findings are particularly significant, considering that diverse correlations between cytokines and anti-Hsp antibodies have been previously reported in autoimmune diseases. For instance, significant positive correlations have been documented in patients with rheumatoid arthritis (RA), including those between anti-Hsp90 IgG levels and IFN-γ (35). Moreover, we recently demonstrated the pathological role of extracellular Hsp70 in epidermolysis bullosa acquisita (EBA), an autoimmune blistering disorder driven by autoantibodies targeting type VII collagen, classified within the group of pemphigoid disease entities (36). Subsequently, we investigated anti-Hsp70 autoantibodies in EBA and found that circulating anti-Hsp70 IgG levels were significantly elevated in EBA patients compared to healthy individuals and positively correlated with serum IFN-γ levels. The pathological significance of anti-Hsp70 IgG antibodies was further confirmed in a mouse model of antibody transfer-induced EBA. In this model, elevated anti-Hsp70 IgG levels were detected, and animals treated with these antibodies exhibited more severe clinical and histological disease manifestations (37). It appears that the relationships observed between the analyzed cytokines and anti-Hsp antibodies in vaccinated individuals differ from those reported in patients with RA and EBA. Thus, it seems that the mRNA vaccine, aside from not altering cytokine levels characteristic of T-helper lymphocyte populations and showing no changes in the levels of anti-Hsp antibodies (21) often associated with the development of autoimmune diseases (19), exhibits correlations between cytokines and anti-Hsp autoantibodies that are atypical for autoimmune conditions such as RA or EBA. Additionally, we have previously found that circulating anti-SARS-CoV-2 antibodies did not cross-react with autoantigens commonly associated with pemphigus or pemphigoid, which are typically targeted by pathogenic autoantibodies in autoimmune blistering diseases (38). Our findings are consistent with the broader consensus on the safety of mRNA-based COVID-19 vaccines concerning autoimmunity. Notably, among COVID-19 patients, completing a two-dose regimen of the vaccine is associated with a reduced risk of conditions such as pemphigoid, Graves’ disease, antiphospholipid antibody syndrome, immune-mediated thrombocytopenia, systemic lupus erythematosus, and other forms of autoimmune arthritis (8).

It is important to note that although blood samples for analysis were collected 3 to 5 weeks after the second vaccine dose, ensuring that the analysis reflects the appropriate phase of the post-vaccination immune response and antibody production, cytokine levels during this period may decrease as the immune response contracts. This could explain why no significant changes were observed between the two analyzed groups. On the other hand, if 3 to 5 weeks is sufficient for cytokine levels to stabilize in vaccinated individuals, we can propose that long-term cytokine disruption does not occur and is unlikely to affect chronic immune activation, which could potentially lead to autoimmune reactions.

## Conclusions

Our findings emphasize the safety of the mRNA COVID-19 vaccine, as it does not affect the secretion of cytokines associated with major T helper cell subpopulations, including Th1, Th2, Th17, and Th22, which are commonly involved in the pathogenesis of autoimmune diseases. Moreover, the relationships observed between the analyzed cytokines and anti-Hsp autoantibodies in vaccinated individuals appear to differ from those seen in patients with RA and EBA. However, further research is needed to assess the safety of COVID-19 vaccination in patients with autoimmune conditions, particularly those in which anti-Hsp autoantibodies are suspected to contribute to disease development.

## Limitations

Our research hypothesis has some limitations. For instance, large-scale follow-up studies are required to validate the immunological effects of COVID-19 vaccination on the onset of autoimmune diseases in a broader population.

## Materials and methods

### Human blood samples and anti-SARS-CoV-2 IgG monitoring

This study included antigen-naive vaccinated individuals (n = 20) who received two doses of the mRNA COVID-19 vaccine encoding the viral spike protein (Pfizer-BioNTech) and age- and sex-matched antigen-naive unvaccinated individuals (anti-SARS-CoV-2 IgG-negative, n = 20) (**Table 1**). The average time interval between the two doses of the mRNA COVID-19 vaccine was 3 weeks, according to Pfizer-BioNTech’s recommendations. Serum samples were collected from blood donors between December 2020 and February 2021, comprising either unvaccinated or vaccinated volunteers with no history of COVID-19. All participants were screened for the presence of anti-SARS-CoV-2 IgG targeting the S1 domain of the viral spike protein using a semiquantitative ELISA test (EUROIMMUN, EI2606-9601-2 G, sensitivity: 94.4%, specificity: 99.6%). In addition, vaccinated participants were assessed for circulating anti-SARS-CoV-2 IgG S1 levels using a quantitative ELISA (Human Anti-SARS-CoV-2 (S) IgG ELISA Kit, A303150) within 3–5 weeks of receiving their second vaccine dose, alongside the analysis of serum cytokines and circulating autoantibodies to Hsps. All serological analyses were performed at a single time point. All vaccinated individuals tested positive for anti-SARS-CoV-2 IgG against the S1 domain, whereas all unvaccinated participants were negative for this marker. The use of human biological material was approved by a bioethics committee at the regional medical chamber in Gdańsk (Poland) and written informed consents were obtained in accordance with the Declaration of Helsinki.

**Table 1:** Demographic characteristics of the participants.

Characteristic	Unvaccinated N = 20^1^	Vaccinated N = 20^1^
Sex		
Female	13 (65%)	16 (80%)
Male	7 (35%)	4 (20%)
Age^2^		
Age	36.55 (±13.22)	39.50 (±10.39)
Ethnicity		
Central European	20 (100%)	20 (100%)

^1^n (%); ^2^Mean (SD).

### Detection of cytokines

Serum levels of various cytokines i.e., IFN-γ, IL-6, IL-22, IL-4, IL-8, IL-10, and IL-17A were measured by commercially available ELISA kits (BioLegend) according to the manufacturer’s protocol.

### Detection of circulating anti-heat shock proteins

Levels of IgG against human Hsp60, Hsp70, and Hsp90 were evaluated in the serum samples by a home-made enzyme-linked immunosorbent assay (ELISA), as described previously (21). In brief, medium-binding 96-well plates were coated overnight at 4°C with full-length recombinant Hsp60 (ab78792, Abcam), Hsp90 (ADI-SPP-770, Enzo Life Science), or previously purified recombinant Hsp70 at a concentration of 0.5 μg/ml in 0.05 M bicarbonate buffer. Following the coating step, the wells were washed and blocked for 90 minutes at room temperature (RT) using 1% bovine serum albumin (BSA) in phosphate-buffered saline (PBS). After washing, the serum samples were diluted in PBS containing 0.1% BSA (05482-100G, Sigma) and added to the wells. Incubation occurred at RT for 90 minutes. Plates were then incubated with horseradish peroxidase (HRP)-conjugated anti-human IgG (096M-4809V, Sigma) secondary antibodies diluted in PBS containing 0.1% BSA, for 60 minutes at RT. The enzymatic reaction was visualized using TMB substrate solution (ab171523, Abcam), and the reaction was halted by the addition of H_2_SO_4_. Optical density (OD) measurements were taken at 450 nm using an ELISA plate reader (VICTOR Multilabel Plate Reader, PerkinElmer). Serum reactivity with the respective Hsps was considered positive if the OD values exceeded the mean BSA reactivity (negative control).

### Statistical analyses

Statistical calculations were carried out using GraphPad Prism 9 (San Diego, CA, USA). To verify whether the data had normal distribution, the Shapiro-Wilk test was used. Data was analyzed by Mann–Whitney U test, Spearman’s rank correlation test The ROUT test was used for outlier identification. P-values less than 0.05 were considered significant.

## Data Availability

The original contributions presented in the study are included in the article/**Supplementary Material**. Further inquiries can be directed to the corresponding author.

## References

[1] Wiersinga WJ Rhodes A Cheng AC Peacock SJ Prescott HC. Pathophysiology, transmission, diagnosis, and treatment of Coronavirus Disease 2019 (COVID-19): a review. JAMA. (2020) 324:782–93. doi: 10.1001/jama.2020.12839 32648899

[2] Wang W Wang CY Wang SI Wei JC. Long-term cardiovascular outcomes in COVID-19 survivors among non-vaccinated population: A retrospective cohort study from the TriNetX US collaborative networks. EClinicalMedicine. (2022) 53:101619. doi: 10.1016/j.eclinm.2022.101619 35971425 PMC9366236

[3] Gupta A Madhavan MV Sehgal K Nair N Mahajan S Sehrawat TS. Extrapulmonary manifestations of COVID-19. Nat Med. (2020) 26:1017–32. doi: 10.1038/s41591-020-0968-3 PMC11972613 32651579

[4] Huang C Wang Y Li X Ren L Zhao J Hu Y. Clinical features of patients infected with 2019 novel coronavirus in Wuhan, China. Lancet. (2020) 395:497–506. doi: 10.1016/s0140-6736(20)30183-5 31986264 PMC7159299

[5] Buszko M Nita-Lazar A Park JH Schwartzberg PL Verthelyi D Young HA. Lessons learned: new insights on the role of cytokines in COVID-19. Nat Immunol. (2021) 22:404–11. doi: 10.1038/s41590-021-00901-9 PMC9317654 33723418

[6] Zhang J. Immune responses in COVID-19 patients: Insights into cytokine storms and adaptive immunity kinetics. Heliyon. (2024) 10:e34577. doi: 10.1016/j.heliyon.2024.e34577 39149061 PMC11325674

[7] Sharma C Bayry J. High risk of autoimmune diseases after COVID-19. Nat Rev Rheumatol. (2023) 19:399–400. doi: 10.1038/s41584-023-00964-y 37046064 PMC10096101

[8] Peng K Li X Yang D Chan SCW Zhou J Wan EYF. Risk of autoimmune diseases following COVID-19 and the potential protective effect from vaccination: a population-based cohort study. EClinicalMedicine. (2023) 63:102154. doi: 10.1016/j.eclinm.2023.102154 37637754 PMC10458663

[9] Liu Y Sawalha AH Lu Q. COVID-19 and autoimmune diseases. Curr Opin Rheumatol. (2021) 33:155–62. doi: 10.1097/bor.0000000000000776 PMC7880581 33332890

[10] Root-Bernstein R. From co-infections to autoimmune disease via hyperactivated innate immunity: COVID-19 autoimmune coagulopathies, autoimmune myocarditis and multisystem inflammatory syndrome in children. Int J Mol Sci. (2023) 24(3):3001. doi: 10.3390/ijms24033001 36769320 PMC9917907

[11] Hromić-Jahjefendić A Mahmutović L Sezer A Bećirević T Rubio-Casillas A Redwan EM. The intersection of microbiome and autoimmunity in long COVID-19: Current insights and future directions. Cytokine Growth Factor Rev. (2024). doi: 10.1016/j.cytogfr.2024.08.002 39179487

[12] Parhiz H AtoChina-Vasserman EN Weissman D. mRNA-based therapeutics: looking beyond COVID-19 vaccines. Lancet. (2024) 403:1192–204. doi: 10.1016/s0140-6736(23)02444-3 38461842

[13] Jung SW Jeon JJ Kim YH Choe SJ Lee S. Long-term risk of autoimmune diseases after mRNA-based SARS-CoV2 vaccination in a Korean, nationwide, population-based cohort study. Nat Commun. (2024) 15:6181. doi: 10.1038/s41467-024-50656-8 39039113 PMC11263712

[14] Kim HJ Kim MH Park SJ Choi MG Chun EM. Autoimmune adverse event following COVID-19 vaccination in Seoul, South Korea. J Allergy Clin Immunol. (2024) 153:1711–20. doi: 10.1016/j.jaci.2024.01.025 38520423

[15] Shan J Hu X Chen T Wang Y Huang B Xin Y. COVID-19 vaccination and the risk of autoimmune diseases: a Mendelian randomization study. Front Public Health. (2024) 12:1322140. doi: 10.3389/fpubh.2024.1322140 38550316 PMC10973840

[16] Guo M Liu X Chen X Li Q. Insights into new-onset autoimmune diseases after COVID-19 vaccination. Autoimmun Rev. (2023) 22:103340. doi: 10.1016/j.autrev.2023.103340 37075917 PMC10108562

[17] Kasperkiewicz M Woodley DT. COVID-19 and autoimmune bullous diseases: Lessons learned. Autoimmun Rev. (2023) 22:103286. doi: 10.1016/j.autrev.2023.103286 36738951 PMC9893837

[18] Ou S Tancrède-Bohin E Alexandre M Ingen-Housz-Oro S Castel M Debarbieux S. Efficacy and safety of anti-COVID-19 vaccination in patients with autoimmune blistering diseases: A French national study. J Am Acad Dermatol. (2024) 90:204–8. doi: 10.1016/j.jaad.2023.08.101 37769901

[19] Tukaj S. Dual role of autoantibodies to heat shock proteins in autoimmune diseases. Front Immunol. (2024) 15:1421528. doi: 10.3389/fimmu.2024.1421528 38903496 PMC11187000

[20] Marino Gammazza A Légaré S Lo Bosco G Fucarino A Angileri F Conway de Macario E. Human molecular chaperones share with SARS-CoV-2 antigenic epitopes potentially capable of eliciting autoimmunity against endothelial cells: possible role of molecular mimicry in COVID-19. Cell Stress Chaperones. (2020) 25:737–41. doi: 10.1007/s12192-020-01148-3 PMC7402394 32754823

[21] Mantej J Bednarek M Sitko K Świętoń M Tukaj S. Autoantibodies to heat shock protein 60, 70, and 90 are not altered in the anti-SARS-CoV-2 IgG-seropositive humans without or with mild symptoms. Cell Stress Chaperones. (2021) 26:735–40. doi: 10.1007/s12192-021-01215-3 PMC8172177 34080135

[22] Kasperkiewicz M Tukaj S. Targeting heat shock proteins 90 and 70: A promising remedy for both autoimmune bullous diseases and COVID-19. Front Immunol. (2022) 13:1080786. doi: 10.3389/fimmu.2022.1080786 36591225 PMC9797581

[23] Tukaj S Sitko K. Heat shock protein 90 (Hsp90) and hsp70 as potential therapeutic targets in autoimmune skin diseases. Biomolecules. (2022) 12(8):1153. doi: 10.3390/biom12081153 36009046 PMC9405624

[24] Sun L Su Y Jiao A Wang X Zhang B. T cells in health and disease. Signal Transduct Target Ther. (2023) 8:235. doi: 10.1038/s41392-023-01471-y 37332039 PMC10277291

[25] Amer SA Al-Zahrani A Imam EA Ishteiwy EM Djelleb IF Abdullh LR. Exploring the reported adverse effects of COVID-19 vaccines among vaccinated Arab populations: a multi-national survey study. Sci Rep. (2024) 14:4785. doi: 10.1038/s41598-024-54886-0 38413637 PMC10899622

[26] Carlini V Noonan DM Abdalalem E Goletti D Sansone C Calabrone L. The multifaceted nature of IL-10: regulation, role in immunological homeostasis and its relevance to cancer, COVID-19 and post-COVID conditions. Front Immunol. (2023) 14:1161067. doi: 10.3389/fimmu.2023.1161067 37359549 PMC10287165

[27] Zhu X Gebo KA Abraham AG Habtehyimer F Patel EU Laeyendecker O. Dynamics of inflammatory responses after SARS-CoV-2 infection by vaccination status in the USA: a prospective cohort study. Lancet Microbe. (2023) 4:e692–703. doi: 10.1016/s2666-5247(23)00171-4 PMC10475695 37659419

[28] Bergamaschi C Terpos E Rosati M Angel M Bear J Stellas D. Systemic IL-15, IFN-γ, and IP-10/CXCL10 signature associated with effective immune response to SARS-CoV-2 in BNT162b2 mRNA vaccine recipients. Cell Rep. (2021) 36:109504. doi: 10.1016/j.celrep.2021.109504 34352226 PMC8299183

[29] Rosati M Terpos E Homan P Bergamaschi C Karaliota S Ntanasis-Stathopoulos I. Rapid transient and longer-lasting innate cytokine changes associated with adaptive immunity after repeated SARS-CoV-2 BNT162b2 mRNA vaccinations. Front Immunol. (2023) 14:1292568. doi: 10.3389/fimmu.2023.1292568 38090597 PMC10711274

[30] Bergamaschi C Pagoni M Rosati M Angel M Tzannou I Vlachou M. Reduced antibodies and innate cytokine changes in SARS-CoV-2 BNT162b2 mRNA vaccinated transplant patients with hematological Malignancies. Front Immunol. (2022) 13:899972. doi: 10.3389/fimmu.2022.899972 35693807 PMC9174567

[31] Cappello F Gammazza AM Dieli F de M Macario AJ. Does SARS-coV-2 trigger stress-inducedAutoimmunity by molecular mimicry? A hypothesis. J Clin Med. (2020) 9(7):2038. doi: 10.3390/jcm9072038 32610587 PMC7408943

[32] Lucchese G Flöel A. SARS-CoV-2 and Guillain-Barré syndrome: molecular mimicry with human heat shock proteins as potential pathogenic mechanism. Cell Stress Chaperones. (2020) 25:731–5. doi: 10.1007/s12192-020-01145-6 PMC7387880 32729001

[33] Yang WC Hwang YS Chen YY Liu CL Shen CN Hong WH. Interleukin-4 supports the suppressive immune responses elicited by regulatory T cells. Front Immunol. (2017) 8:1508. doi: 10.3389/fimmu.2017.01508 29184551 PMC5694475

[34] Tukaj S Kaminski M. Heat shock proteins in the therapy of autoimmune diseases: too simple to be true? Cell Stress Chaperones. (2019) 24:475–9. doi: 10.1007/s12192-019-01000-3 PMC6527538 31073900

[35] Mantej J Polasik K Piotrowska E Tukaj S. Autoantibodies to heat shock proteins 60, 70, and 90 in patients with rheumatoid arthritis. Cell Stress Chaperones. (2019) 24:283–7. doi: 10.1007/s12192-018-0951-9 PMC6363621 30465159

[36] Tukaj S Mantej J Sitko K Bednarek M Zillikens D Ludwig RJ. Evidence for a role of extracellular heat shock protein 70 in epidermolysis bullosa acquisita. Exp Dermatol. (2022) 31:528–34. doi: 10.1111/exd.14495 34741567

[37] Tukaj S Mantej J Sitko K Zillikens D Ludwig RJ Bieber K. Pathological relevance of anti-hsp70 IgG autoantibodies in epidermolysis bullosa acquisita. Front Immunol. (2022) 13:877958. doi: 10.3389/fimmu.2022.877958 35514963 PMC9065281

[38] Kasperkiewicz M Bednarek M Tukaj S. Case report: circulating anti-SARS-CoV-2 antibodies do not cross-react with pemphigus or pemphigoid autoantigens. Front Med (Lausanne). (2021) 8:807711. doi: 10.3389/fmed.2021.807711 34988105 PMC8720918

