# Supplementary material for: The impact of the mRNA COVID-19 vaccine on the Th-like cytokine profile in individuals with no history of COVID-19: insights into autoimmunity targeting heat shock proteins
Source: Front Immunol. 2025 Mar 14;16:1549739. doi: 10.3389/fimmu.2025.1549739 (PMC11949786; doi:10.3389/fimmu.2025.1549739)
Supplement: Supplementary file 1 [file DataSheet1.pdf]

(A)

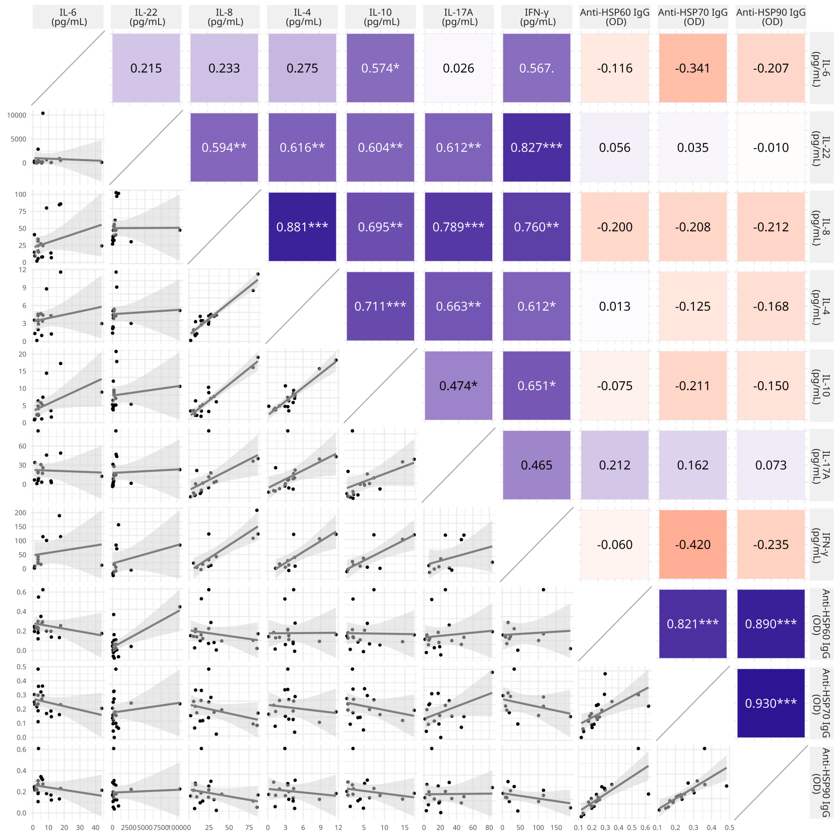

(B)

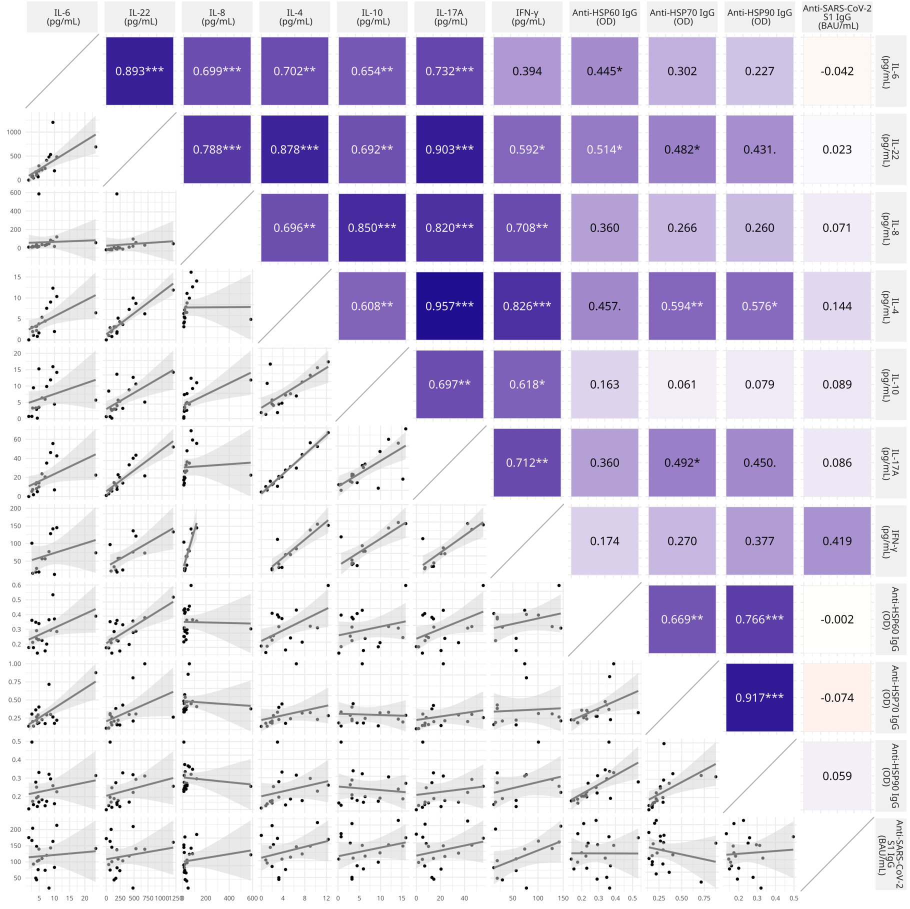

Supplementary Data 1. Scatter plots and correlation analyses illustrating the relationships between serum levels of IL-6, IL-22, IL-4, IL-8, IL-10, IL-17A, IFN-γ, and circulating anti-Hsp IgGs or anti-SARS-CoV-2 IgG S1, presented as Spearman's rank correlation coefficients. Comparisons were conducted within the groups: (A) anti-SARS-CoV-2 IgG-negative (Unvaccinated) and (B) anti-SARS-CoV-2 IgG-positive (Vaccinated). Correlation coefficients (r values) are shown within the heatmap boxes, with statistically significant correlations marked with asterisks (*P < 0.05, **P < 0.01, ***P < 0.001).
